# The effects of conditioned media generated by polarized macrophages on the cellular behaviours of bone marrow mesenchymal stem cells

**DOI:** 10.1111/jcmm.13431

**Published:** 2017-11-06

**Authors:** Xiao‐Tao He, Xuan Li, Yuan Yin, Rui‐Xin Wu, Xin‐Yue Xu, Fa‐Ming Chen

**Affiliations:** ^1^ State Key Laboratory of Military Stomatology Department of Periodontology National Clinical Research Center for Oral Diseases and Shaanxi Engineering Research Center for Dental Materials and Advanced Manufacture School of Stomatology Fourth Military Medical University Xi'an China

**Keywords:** macrophage polarization, cell culture, conditioned medium, mesenchymal stem cell, cell differentiation, cell sheet engineering

## Abstract

Macrophages (Mφs) are involved in a variety of physiological and pathological events including wound healing and tissue regeneration, in which they play both positive and negative roles depending on their polarization state. In this study, we investigated the cellular behaviours of bone marrow mesenchymal stem cells (BMMSCs) after incubation in different conditioned media (CMs) generated by unpolarized Mφs (M0) or polarized Mφs (M1 and M2). Mφ polarization was induced by stimulation with various cytokines, and CMs were obtained from *in vitro* Mφ cultures termed CM0, CM1 and CM2 based on each Mφ phenotype. We found that CM1 supported the proliferation and adipogenic differentiation of BMMSCs, whereas CM0 had a remarkable effect on cell osteogenic differentiation. To a certain degree, CM2 also facilitated BMMSC osteogenesis; in particular, cells incubated with CM2 exhibited an enhanced capacity to form robust stem cell sheets. Although incubation with CM1 also increased production of extracellular matrix components, such as fibronectin, COL‐1 and integrin β1during sheet induction, the sheets generated by CM2‐incubated cells were thicker than those generated by CM1‐incubated cells (*P* < 0.001). Our data suggest that each Mφ phenotype has a unique effect on BMMSCs. Fine‐tuning Mφ polarization following transplantation may serve as an effective method to modulate the therapeutic potential of BMMSCs.

## Introduction

Translation of stem cells and other regenerative paradigms from bench to bedside requires an understanding of host responses to outside cellular and/or material transplants 1. Both *in vitro* and *in vivo* conditions can influence the cellular behaviours of cellular materials as well as their therapeutic potential following transplantation 2, 3, 4. In particular, innate immune responses to transplant‐based strategies, especially those arising from macrophages (Mφs), have received much attention in recent years in fields ranging from tissue engineering to *in situ* tissue regeneration 5, 6. Most previous approaches have focused on minimizing immune responses to bioengineered devices. However, it is now well recognized that an immune response is almost inevitable following transplantation; indeed, regenerative medicine is an approach centred on both pro‐ and anti‐inflammatory responses 1. Accordingly, it has been proposed that desirable regenerative paradigms should instead accommodate and facilitate ideal involvement of the host immune response to promote positive regenerative outcomes 1, 6, 7.

Mφs play critical roles in immune responses, and their phenotype and function have recently been demonstrated as key and, in some cases, determinant factors in downstream outcomes 1, 8. In response to different microenvironmental stimuli, resting/unpolarized Mφs (M0) can be activated into distinct states (phenotypes), with each function having a unique role during physiological or pathological events 9. In the most simplistic form, polarized Mφs are divided into pro‐inflammatory M1 Mφs and anti‐inflammatory M2 Mφs (reviewed in 1, 8). In general, M1‐polarized Mφs secrete proinflammatory cytokines such as interleukin‐6 (IL‐6), tumour necrosis factor‐α (TNF‐α) and inducible nitric oxide synthase (iNOS) to exert an inflammatory response, whereas M2‐polarized Mφs produce anti‐inflammatory cytokines such as IL‐10 and arginase‐1 (Arg‐1) to suppress inflammation and induce regulatory activity to modulate wound healing and tissue regeneration 10, 11, 12. It has also been reported that the balance between M1 and M2 Mφs is critical in pathogen phagocytosis, apoptotic cell clearance and injured tissue healing/remodelling 10, 11, 13, 14.

Mφs exhibit great functional diversity. Their polarization state can be adjusted in response to microenvironmental cues 12, 15, 16, and emerging evidence indicates that fine‐tuning the balance of Mφ phenotypes can promote angiogenesis and vascularization in tissue engineering constructs 14, 17, 18, 19, 20. Although such modulation, at least in part, contributes to the multifaceted effects of polarized Mφs on the mesenchymal stem cells (MSCs) involved, cell–cell contacts between stem cells and Mφs remain largely unexplored. Previous studies indicate that osteal tissue Mφs can regulate osteoblast function and promote fracture repair 21, 22, and there is evidence that activated monocytes/Mφs can promote osteogenic differentiation of MSCs 23, 24, 25. Nonetheless, different Mφ phenotypes may exert different cellular functions. Importantly, the factors used to stimulate Mφ polarization may also influence stem cells, and such influences have been ignored in the aforementioned investigations.

In this study, Mφ polarization was induced by various cytokine stimuli 26, and conditioned media (CMs) derived from polarized Mφs (CM0, CM1 and CM2) was used to investigate the impact of Mφs on the properties of bone marrow mesenchymal stem cells (BMMSCs). It should be noted that stimulating factors were completely removed before the supernatants of polarized Mφs were collected for CM production 27, 28. In addition, the application of CM in investigations can prevent changes in Mφ polarization when directly co‐cultured with BMMSCs, as there is evidence that MSCs exert significant effects on macrophages during *in vitro* culture 29. Given that cell sheets generated by stem cell cultures are widely used as cellular materials in regenerative design, we also investigated the influence of various Mφs on matrix production and stem cell sheet formation. Our goal was to clarify the multifaceted effects of polarized Mφs on cell behaviour during *in vitro* culture, which may provide useful information for future transplant‐based strategies that fine‐tune Mφ polarization towards an appropriate regenerative outcome.

## Materials and methods

### Isolation and culture of BMMSCs

The isolation and use of mouse BMMSCs were approved by the Animal Care Committee of the Fourth Military Medical University, Xi'an, China. Based on a previously reported procedure 30, bone marrow cells were flushed from the bone cavity of femurs and tibias using complete medium, *that is* alpha‐minimal essential medium (α‐MEM; Invitrogen, Carlsbad, CA, USA) supplemented with 20% foetal bovine serum (FBS; Hangzhou Sijiqing Biological Engineering Materials, Zhejiang, China), 0.292 mg/ml glutamine (Invitrogen) and 1% penicillin and streptomycin (both from Gibco BRL, Gaithersburg, MD, USA). A sample of 1.5 × 10^7^ cells was seeded in 100‐mm culture dishes (Nest Biotechnology, Wuxi, China) and cultured in a humidified atmosphere of 5% CO_2_ at 37°C; the medium was changed every 3 days. The cells were passaged to approximately 80% confluence by digestion with 0.25% trypsin (trypsin‐EDTA, Invitrogen) for 3 min. and then expansion in the different CMs generated by cells with different Mφ phenotypes (M0, M1 and M2).

### Mφ polarization and preparation of CMs

Murine‐derived Mφ RAW 264.7 cells were incubated in 100‐mm culture dishes containing normal complete medium (α‐MEM supplemented with 20% FBS, 0.292 mg/ml glutamine, and 1% penicillin and streptomycin) at 37°C in a humidified atmosphere of CO_2_. To induce Mφ polarization 26, 10 ng/ml interferon‐gamma (IFN‐γ) and 200 ng/ml lipopolysaccharide (LPS) were added to the cultures to generate M1‐polarized Mφs, and 20 ng/ml IL‐4 was used to generate M2‐polarized Mφs (all cytokines were from PeproTech, Princeton, NJ, USA). Mφs incubated in parallelly designed dishes supplemented with phosphate‐buffered saline (PBS) were considered M0‐unpolarized Mφs. After 24 hrs, the cells were washed twice with PBS to ensure that the cytokines used in the cultures were completely removed, and the medium was then replaced with complete culture medium. After culturing for another 24 hrs, the medium supernatants were collected and centrifuged at 1950 g for 20 min. and then filtered through 0.22‐μm‐pore filters (Millipore, Billerica, MA, USA) to remove cells and debris. CMs generated by M0‐unpolarized, M1‐polarized and M2‐polarized cell cultures were termed CM0, CM1 and CM2, respectively. The cellular behaviours of BMMSCs cultured in CM0, CM1 and CM2 were investigated, and cells cultured in normal complete medium (Norm) were used as the control.

### Identification of polarized Mφs

#### Flow cytometric analysis

After stimulation for 24 hrs, the immunophenotype of RAW 264.7 cells was evaluated by flow cytometry, as described previously 31. Briefly, 1 × 10^6^ M0‐unpolarized, M1‐polarized or M2‐polarized Mφs were trypsinized and washed twice with cold PBS. The cell suspension was then divided into sterile Eppendorf tubes and blocked with 2% anti‐mouse CD16/32 (Biolegend, San Diego, CA, USA) on ice for 10 min. The cells were then washed twice and incubated with the following antibodies for half an hour in the dark: PE anti‐mouse CD86 and APC anti‐mouse CD206 (both from Biolegend). Cells that were not pretreated with any antibody were used as blank controls. The cells were washed twice to remove excess antibodies, resuspended in 400 μl PBS containing 3% FBS and analysed with a Beckman Coulter Epics XL cytometer (Beckman Coulter, Fullerton, CA, USA).

#### Quantitative real‐time polymerase chain reaction (qRT‐PCR)

Real‐time reverse transcriptional polymerase chain reaction was performed as previously described 32, 33. The primers employed in the current study are shown in Table 1. The housekeeping gene β*‐actin* was used for normalization.

**Table 1:** Primer sequences used in this study

Gene	Full name	Primer	Sequences (5′‐3′)
*iNOS*	Inducible nitric oxide synthase	Forward	CAAGCTGAACTTGAGCGAGGA
		Reverse	TTTACTCAGTGCCAGAAGCTGGA
*TNF‐α*	Tumour necrosis factor‐α	Forward	TATGGCCCAGACCCTCACA
		Reverse	GGAGTAGACAAGGTACAACCCATC
*CCR7*	Chemokine receptor type‐7	Forward	TGGTCAGTGCCCAAGTGGAG
		Reverse	TCAAAGTTGCGTGCCTGGAG
*Arg‐1*	Arginine‐1	Forward	AGCTCTGGGAATCTGCATGG
		Reverse	ATGTACACGATGTCTTTGGCAGATA
*CD206*	CD206	Forward	AGCTTCATCTTCGGGCCTTTG
		Reverse	GGTGACCACTCCTGCTGCTTTAG
*IL‐10*	Interleukin‐10	Forward	GCCAGAGCCACATGCTCCTA
		Reverse	GATAAGGCTTGGCAACCCAAGTAA
*Adiponectin*	Adiponectin	Forward	GAGAGAAAGGAGATGCAGGT
		Reverse	GAACGCTGAGCGATACACAT
*PPAR‐γ*	Peroxisome proliferator activated receptor‐γ2	Forward	AGCTCCAAGAATACCAAAGT
		Reverse	ACCCTTGCATCCTTCACAAG
*Runx2*	Runt‐related transcription factor‐2	Forward	GACTGTGGTTACCGTCATGGC
		Reverse	ACTTGGTTTTTCATAACAGCGGA
*ALP*	Alkaline phosphatase	Forward	CTTCTTGCTGGTGGAAGGA
		Reverse	AAAACGTGGGAATGATCAGC
*SP7*	SP7 transcription factor‐2	Forward	GGGGAAAGGAGGCACAAAG
		Reverse	GTGAGGGAAGGGTGGGTAGTC
*OCN*	Osteocalcin	Forward	CTGACAAAGCCTTCATGTCCAA
		Reverse	GCGCCGGAGTCTGTTCACTA
*COL‐1*	Collagen‐1	Forward	GCTGGAGTTTCCGTGCCT
		Reverse	GACCTCGGGGACCCATTG
*Fibronectin*	Fibronectin	Forward	AATCACAGTAGTTGCGGCAGGAGA
		Reverse	TCTGTCCCAGGCAGGAGATTTGTT
*Integrin β1*	Integrin β1	Forward	CGCAGAACAATAGGTGCTGAAATTAC
		Reverse	TGACACTGAGAACCACAAACGGC
*β‐actin*	β‐actin	Forward	CTCTTTTCCAGCCTTCCTTCTT
		Reverse	GAGGTCTTTACGGATGTCAACG

#### Fluorescence staining and imaging

For fluorescence staining, cell samples were fixed in 4% paraformaldehyde for 30 min at room temperature and washed three times with PBS (3 min each). The cells were then permeabilized and blocked with 2% normal donkey serum in 0.01 M PBS containing 0.3% Triton X‐100, 0.02% sodium azide and 0.12% carrageenan (pH 7.4, all reagents were purchased from Sigma‐Aldrich, St. Louis, MO, USA) for 1 hr at room temperature. Samples were subsequently incubated overnight at 4°C with the primary antibody. For double immunofluorescence, samples were incubated with a mixture of two primary antibodies followed by a mixture of the two respective secondary antibodies, Alexa 488 donkey anti‐mouse IgG (1:500, A21202, Invitrogen) or Alexa 594 donkey anti‐rabbit IgG (1:500, A21207, Invitrogen), and counterstained with DAPI (4′,6‐diamidino‐2‐phenylindole, 1:5000, Invitrogen). The following primary antibodies were used: mouse anti‐TNF‐α IgG (1:300, sc‐52746, Santa Cruz Biotechnology, San Diego, CA, USA), mouse anti‐IL‐10 IgG (1:300, sc‐57244, Santa Cruz Biotechnology) and rabbit anti‐β‐actin IgG (CW0096A, ComWin Biotech, Beijing, China; 1:2000). Confocal images were obtained using a confocal laser microscope (FV1000; Olympus, Tokyo, Japan), and digital images were captured with FluoView 1000 (Olympus).

### Effects of CMs generated by polarized Mφs on BMMSC proliferation

#### CCK‐8 assays

The proliferative capacity of isolated BMMSCs incubated in different conditioned media (CM0, CM1 and CM2) or complete medium (Norm) was quantified using a Cell Counting Kit‐8 assay Kit (7 Sea Biotech, Shanghai, China). According to the manufacturer's instructions, BMMSCs (3 × 10^3^ cells per well) were seeded in a 96‐well plate (Costar, Cambridge, MA, USA) with 200 μl complete medium containing 20% FBS. After cell adhesion, the medium was changed to CM0, CM1, CM2 or Norm. The CCK‐8 assay was performed every day at a fixed time‐point during the 7‐day incubation period, and the medium was refreshed every other day. Each day, 20 μl CCK‐8 solution was added to each test well, followed by incubation at 37°C for 3 hrs. The supernatant was then transferred to another 96‐well plate, and the optical density (OD) at 450 nm was immediately determined using a microplate reader (ELx800, BioTek Instruments, Highland Park, USA).

#### Colony‐forming assay

Bone marrow mesenchymal stem cells were plated in 100‐mm culture dishes at a density of 2000 cells per dish. After 24 hrs of pre‐incubation, the culture medium was replaced with CM0, CM1, CM2 or Norm; the medium was refreshed every 4 days. On day 14, the cells were fixed in 4% paraformaldehyde for 30 min. and then stained with 1% toluidine blue (Beyotime, Shanghai, China) for 15 min. at room temperature. Surface staining was removed by rinsing three times with double‐distilled water, and the cells were imaged under a stereomicroscope. The number of colony‐forming units (CFUs, ≥50 cells) was quantified for statistical analysis.

#### Edu (5‐ethynyl‐2′‐deoxyuridine) incorporation assay

Bone marrow mesenchymal stem cells were seeded in 6‐well culture plates (NEST Biotechnology) at a density of 4 × 10^5^ cells per well and cultured with CM0, CM1, CM2 or Norm. When cells reached 80% confluence, an EdU incorporation assay was performed using a keyFluor594 Click‐iT EdU Kit (KeyGEN BioTECH, Nanjing, China). Cells were first incubated with medium supplemented with 50 μM EdU for 3 hrs and fixed in 4% polyformaldehyde for 30 min. The cells were then permeabilized with 0.5% Triton X‐100 and subsequently blocked with 3% bovine serum albumin (BSA) in PBS for 2 hrs. EdU detection was performed following the manufacturer's instructions. EdU‐labelled cells were visualized under an FV1000 confocal laser scanning microscope (Olympus).

### Effects of polarized Mφ CMs on BMMSC differentiation

#### Cell differentiation assays

To assess the adipogenic ability of BMMSCs, the growth medium was changed to adipogenic medium (Norm, CM0, CM1 or CM2 medium supplemented with 0.5 mM 3‐isobutyl‐1‐methylxanthine, 1 μM dexamethasone, 0.1 mM indomethacin and 10 μg/ml insulin, all from Sigma‐Aldrich). Each medium was refreshed every other day. After 7 days of induction, the cells were fixed in paraformaldehyde and stained with Oil Red O (Sigma‐Aldrich) for 30 min. at room temperature. Lipid droplets were then dissolved in 200 μl isopropanol, and OD values were quantitatively measured at 560 nm to assess the adipogenic potential of the BMMSCs. Cell samples were collected after 7 days of adipogenic induction for qRT‐PCR and Western blotting.

To assess the ability of BMMSCs to form mineralized nodules, cells were seeded at a density of 2 × 10^5^ cells per well in 12‐well culture plates with Norm, CM0, CM1 or CM2. When the cells reached 80% confluence, the media were changed to osteoinductive media (Norm, CM0, CM1 or CM2 supplemented with 50 μg/ml vitamin C, 10 nM dexamethasone and 10 mM β‐glycerophosphate, all purchased from Sigma‐Aldrich); each medium was refreshed every other day. After 2 weeks of osteogenic induction, the cells were fixed in 4% paraformaldehyde for 30 min. and stained with 0.1% Alizarin Red S (Sigma‐Aldrich) for 1 hr at room temperature. After washing twice with double distilled water, surface staining was imaged under a stereomicroscope. For quantification, the mineralized nodules were dissolved in 200 μl 2% cetylpyridinium chloride for 2 hrs at room temperature, and the OD values of the solutions were measured at 560 nm. Cell samples after 7 days of osteogenic induction were collected for alkaline phosphatase (ALP) staining, ALP activity assessment and qRT‐PCR analysis (as described in section Quantitative real‐time polymerase chain reaction (qRT‐PCR)).

#### Western blotting

Western blot analysis was performed as previously described 32, 34. The following primary antibodies were used: antibodies targeting PPAR‐γ (ab59324, Abcam, Cambridge, UK; 1:500) and β‐actin for adipogenic samples; antibodies targeting ALP (ab108337, Abcam; 1:500), Runx2 (#12556, Cell Signaling Technology, Danvers, MA, USA; 1:1000), OCN (ab93876, Abcam; 1:500) and SP7 (av31622, Sigma‐Aldrich; 1:2000) for osteogenic samples. Horseradish peroxidase (HRP)‐conjugated anti‐rabbit (CW0103, ComWin Biotech; 1:40,000) or anti‐mouse (CW0102, ComWin Biotech; 1:40,000) secondary antibodies were used. To quantify protein levels, the grey values of the blots in scanned images were measured using ImageJ Plus software (National Institute of Health, Bethesda, MD, USA), and the grey value of each target protein was normalized to that of β‐actin before comparison.

#### ALP staining and activity

Osteogenic induction of BMMSCs was performed as described in section Cell differentiation assays. After 7 days of induction, the cell culture supernatants were collected and centrifuged at 1460 g for 15 min. at 4°C to remove cell residues and other impurities. ALP activity was then determined using an ALP activity detection kit (Jiancheng Bioengineering, Nanjing, China) according to the manufacturer's instructions. The cell samples were washed three times with PBS and fixed in 4% paraformaldehyde for 30 min. ALP staining was then performed according to the BCIP/NBT Alkaline Phosphatase Color Development Kit protocol (Beyotime Institute of Biotechnology, Haimen, China).

### Effects of polarized Mφ CMs on cell sheet formation

#### Cell sheet formation

Bone marrow mesenchymal stem cells were seeded in 12‐well dishes in CM0, CM1, CM2 or Norm at a density of 5 × 10^5^ cells per well. Each CM was mixed with an equal volume of complete medium for the following experiment. When the cells reached 80% confluence, all media were changed to cell‐sheet induction medium (CMs or Norm supplemented with 50 μg/ml vitamin C) for cell sheet formation, as previously described 32. The medium was refreshed every other day. After 7 days of induction, the cell sheets were harvested using sterile tweezers and prepared for haematoxylin and eosin (HE) staining, qRT‐PCR, Western blotting and fluorescent staining to evaluate sheet formation and extracellular matrix (ECM) production by BMMSCs.

#### Haematoxylin and eosin (HE) staining

Haematoxylin and eosin staining was performed, and the stained sheets were analysed using previously reported methods 35. Briefly, cell sheets were fixed in 4% paraformaldehyde for 12 hrs. The obtained cell sheets were then rinsed, dehydrated, embedded in paraffin and cut into 5‐μm‐thick sections using a rotary microtome (Reichert‐Jung 820, Cambridge Instruments GmbH, Nussloch, Germany) and stained with HE to visualize the thickness using Photoshop CS 5.0 software.

#### ECM production by BMMSCs in different CMs

Production of ECM proteins (fibronectin, COL‐1 and integrin β1) in BMMSC sheets was determined by Western blotting (as described in section Western blotting). The following primary antibodies were used: mouse anti‐fibronectin (ab32419, Abcam; 1:1000), ‐Collagen‐1 (COL‐1, ab90395, Abcam; 1:1000) and ‐integrin β1 (ab179471, Abcam; 1:1000). qRT‐PCR was performed as described in section Quantitative real‐time polymerase chain reaction (qRT‐PCR). The primers employed in the present study are provided in Table 1. Fibronectin, COL‐1 and integrin β1 protein expression before detachment from 12‐well plates was also detected by immunofluorescence analysis (as described in section Fluorescence staining and imaging).

### Statistical analyses

All results are presented as the means ± standard deviations (SD) of at least three independent experiments for each cell line. Data were analysed by one‐way analysis of variance (anova) and Tukey's post‐test or two‐way anova using GraphPad Prism 5.0 software (San Diego, CA, USA). Statistical significance was considered at *P* < 0.05, 0.01 and 0.001.

## Results

### Characterization of polarized Mφs

**Figure 1:**
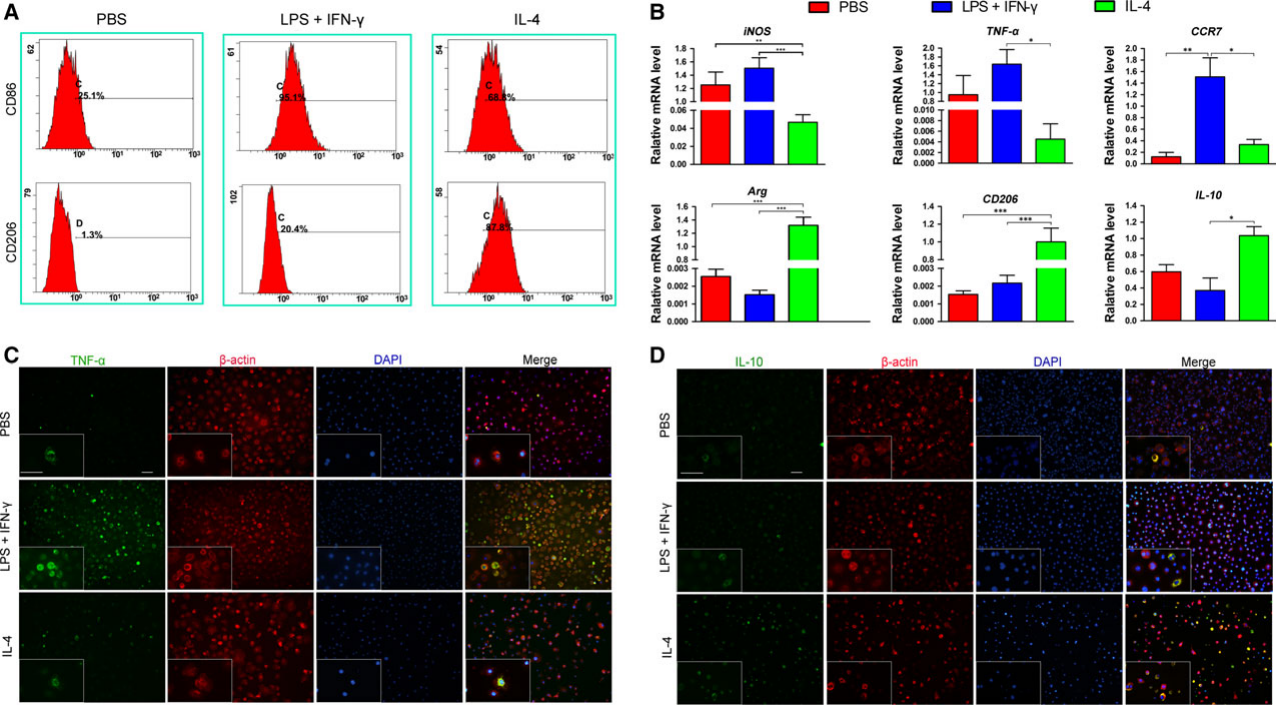
Characterization of Mφ phenotypes following PBS (control), LPS plus IFN‐γ or IL‐4 stimulus. (**A**) Expression of CD86 (M1 marker) and CD206 (M2 marker) in polarized RAW 264.7 cells analysed by flow cytometry. (**B**) Gene expression in polarized RAW 264.7 cells analyzed by qRT‐PCR (*iNOS*,*TNF‐*α and *CCR7* were used as M1‐polarized markers; *Arg*,*CD206* and *IL‐10* were used as M2‐polarized markers; normalized to β*‐actin*); data are shown as the mean ± SD; *P < 0.05, **P < 0.01 and ***P < 0.001 represent significant differences between the indicated columns. Immunofluorescent staining of M1 marker TNF‐α (**C**) and M2 marker IL‐10 (**D**) in polarized RAW 264.7 cells (scale bar: 50 μm).

Mφ RAW 264.7 cells polarized to different Mφ phenotypes with the addition of specific cytokines (IL‐4 or IFN‐γ plus LPS). Cells treated with PBS were used as an unpolarized phenotype (M0). Flow cytometric analysis was employed to detect surface markers of the Mφ phenotypes. CD86 (M1 surface marker) was highly expressed by cells polarized with IFN‐γ plus LPS, although expression was also detected in cells polarized with IL‐4 (Fig. 1A). Similarly, flow cytometric analysis revealed stronger CD206 expression in cells polarized using the IL‐4 stimulus (M2 surface marker) than in those polarized with PBS or IFN‐γ (Fig. 1A).

Previous research has identified the specific markers expressed by each Mφ phenotype 10. *iNOS, TNF‐*α and *CCR7*, known as M1‐specific markers, were significantly up‐regulated in cells polarized with IFN‐γ plus LPS compared with those polarized with IL‐4 (*P* < 0.05 or 0.01, Fig. 1B). In addition, expression of *CCR7* in cells polarized with IFN‐γ plus LPS was also significantly higher than in those polarized with PBS (*P* < 0.05, Fig. 1B). Moreover, there was no significant difference in expression of *TNF‐*α or *CCR7* between cells exposed to PBS and those exposed to IL‐4 (Fig. 1B). In addition, the expression levels of *Arg, CD206* and *IL‐10*, known as M2 specific markers, were significantly higher in cells exposed to IL‐4 than in those exposed to PBS or IFN‐γ plus LPS (*P* < 0.05 or 0.001, Fig. 1B), with the exception of *IL‐10* between cells exposed to PBS and those exposed to IL‐4 (Fig. 1B).

Immunofluorescence was used to confirm that cells stimulated with IFN‐γ plus LPS or IL‐4 fused into M1‐polarized or M2‐polarized Mφs, respectively. Consistent with the results of PCR, TNF‐α staining was much stronger in cells polarized with IFN‐γ plus LPS than in those polarized with IL‐4 or PBS (Fig. 1C). Similarly, cells polarized using IL‐4 exposure exhibited much stronger staining of IL‐10 compared with those polarized with IFN‐γ or PBS (Fig. 1D).

### Effects of polarized Mφ CMs on BMMSC proliferation

**Figure 2:**
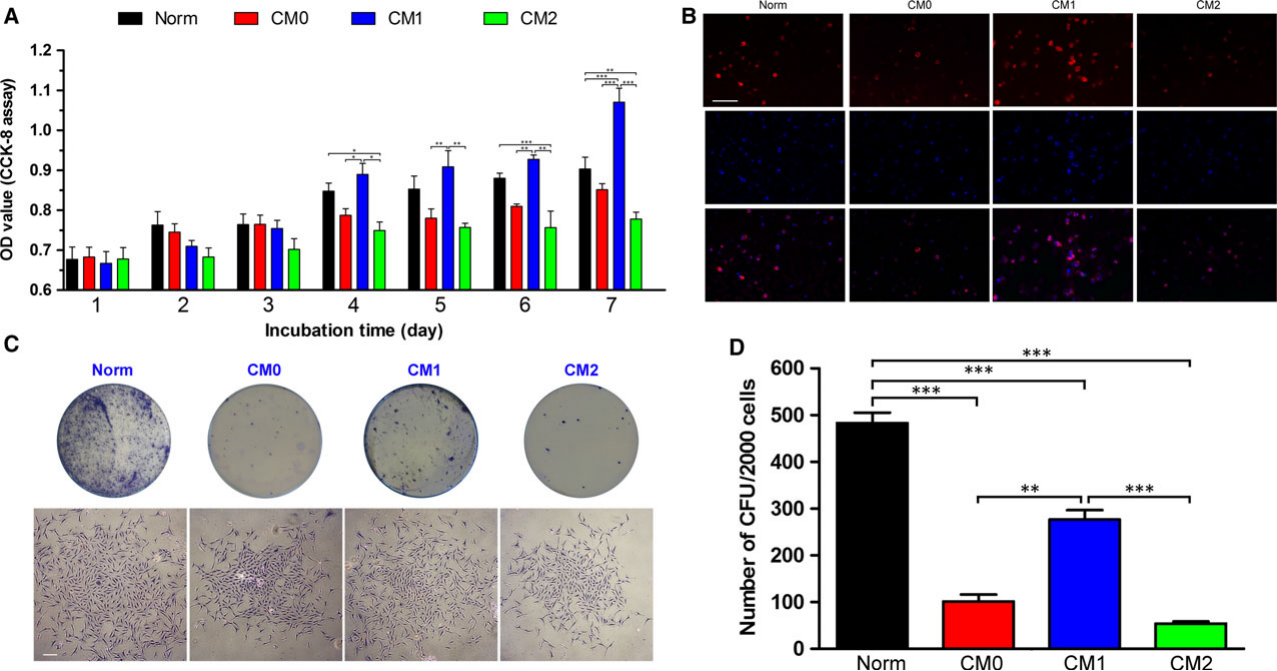
Effects of CMs generated by unpolarized Mφs (CM0) and polarized Mφs (CM1 and CM2) on bone marrow mesenchymal stem cell (BMMSC) proliferation; normal complete medium (Norm) was used as a control. (**A**) Proliferative capacity of BMMSCs in different cultures evaluated by Cell Counting Kit‐8 (CCK‐8) assays during a 7‐day incubation period. (**B**) Proliferative capacity of BMMSCs in different cultures determined by EdU incorporation assays; representative images showing EdU‐positive cells (red fluorescence; scale bar: 100 μm). (**C**) Representative images of colony‐forming units (CFUs) (top) and a single CFU (bottom) formed by BMMSCs in different cultures on day 14 (scale bar: 250 μm). (**D**) Quantitative analysis of the number of CFUs formed by BMMSCs in different cultures; data are shown as the mean ± SD; **P < 0.01 and ***P < 0.001 represent significant differences between the indicated columns.

The proliferative capacity of BMMSCs cultured in CMs or Norm was analysed *via* CCK‐8 assays. The ascending trend of the cell growth histogram for BMMSCs incubated in CM1 was significantly higher than that for BMMSCs incubated in CM0 or CM2 from day 4 after passage (*P* < 0.05 or 0.01, Fig. 2A). After 7 days of incubation, the OD value of BMMSCs incubated in CM1 was significantly higher than that of cells incubated in Norm (*P* < 0.05, Fig. 2A). A significant difference between cells cultured in Norm and CM2 was also observed following the last 4‐day culture period (*P* < 0.01 or 0.001, Fig. 2A). Consistent with the results of CCK‐8 assays, the results of EdU incorporation assays revealed more EdU‐positive cells after incubation in CM1 than in Norm (Fig. 2B), suggesting that these cells had greater proliferative potential than cells incubated in CM0 or CM2.

When BMMSCs were cultured in 100‐mm dishes at a density of 3000 cells/dish in CM0, CM1, CM2 or Norm medium, all cells tested demonstrated the ability to form colony units (Fig. 2C). However, cells cultured in Norm exhibited the highest degree of CFU formation among the four tested groups (*P* < 0.001, Fig. 2D). Moreover, the number of CFUs formed by cells incubated in CM1 was significantly higher than that formed by cells incubated in CM0 or CM2 (*P* < 0.01 or 0.001, Fig. 2D).

### Effects of polarized Mφ CMs on BMMSC adipogenic differentiation

**Figure 3:**
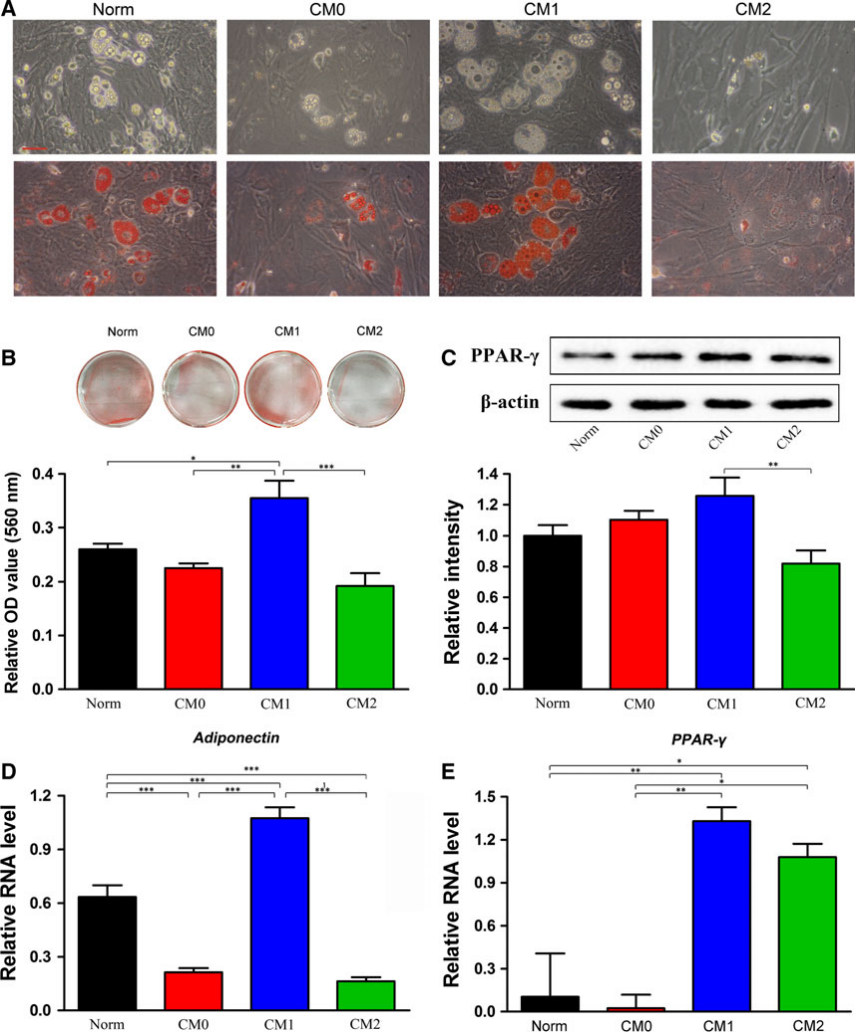
Effects of CMs generated by different phenotypes of Mφs on adipogenic differentiation of bone marrow mesenchymal stem cells (BMMSCs); Norm was used as a control. (**A**) Representative images of lipid droplets before and after Oil Red O staining were observed after 7 days of induction. Scale bar: 50 μm. (**B**) General view of Oil Red O staining and quantitative analysis of lipid droplets formed by BMMSCs in different CM‐ and Norm‐based adipogenic media. (**C**) Expression of the PPAR‐γ protein after 7 days of adipogenic induction determined by Western blotting. The analysis was based on mean grey values normalized to β‐actin. Representative bands are shown at the top, and a summary of the data is shown at the bottom. (**D,E**) Relative mRNA expression (normalized to β*‐actin*) of *adiponectin* (**D**) and *PPAR‐*γ (**E**). Data are expressed as the mean ± SD; *P < 0.05, **P < 0.01 and ***P < 0.001 represent significant differences between the indicated columns.

The adipogenic capacity of BMMSCs cultured in CMs or Norm was evaluated under adipo‐inductive conditions. The results indicated that BMMSCs cultured in either CM‐ or Norm‐based adipogenic medium were capable of undergoing adipogenic differentiation and forming microscopic Oil Red O‐positive lipid droplets following a 7‐day induction (Fig. 3A). BMMSCs induced in CM1‐based inductive medium generated more lipid droplets than those induced in CM0‐ or CM2‐based inductive medium (Fig. 3A). Quantitative analysis of the lipid droplets in the cells also revealed the highest OD value for cells induced in CM1‐based adipogenic medium among the four tested groups (*P* < 0.05, 0.01 or 0.001, Fig. 3B).

PPAR‐γ, a transcription factor essential for adipogenesis, was analysed by both Western blotting and qRT‐PCR. Western blot analysis showed that the PPAR‐γ protein level in cells induced in CM1‐based adipogenic medium was significantly up‐regulated compared with those induced in CM2‐based inductive medium (*P* < 0.05, Fig. 3C). Although expression of PPAR‐γ in CM0 and Norm was lower than that in CM1, no statistically significant difference among the three groups was observed (Fig. 3C). Furthermore, qRT‐PCR analysis of *PPAR‐*γ (an early marker of adipogenesis) and *adiponectin* (a late marker of adipogenesis) showed that CM1 resulted in the greatest BMMSC adipogenic differentiation potential (*P* < 0.01 or 0.001, Fig. 3D and E). In addition, cells cultured in CM2‐based inductive medium showed a significantly higher level of *PPAR‐*γ gene expression than cells cultured in CM0‐ or Norm‐based medium (*P* < 0.05, Fig. 3E). However, expression of *adiponectin* was significantly lower in cells cultured in Norm‐based medium compared to those cultured in CM2‐based inductive medium (*P* < 0.001, Fig. 3D).

### Effects of polarized Mφ CMs on BMMSC osteogenic differentiation

**Figure 4:**
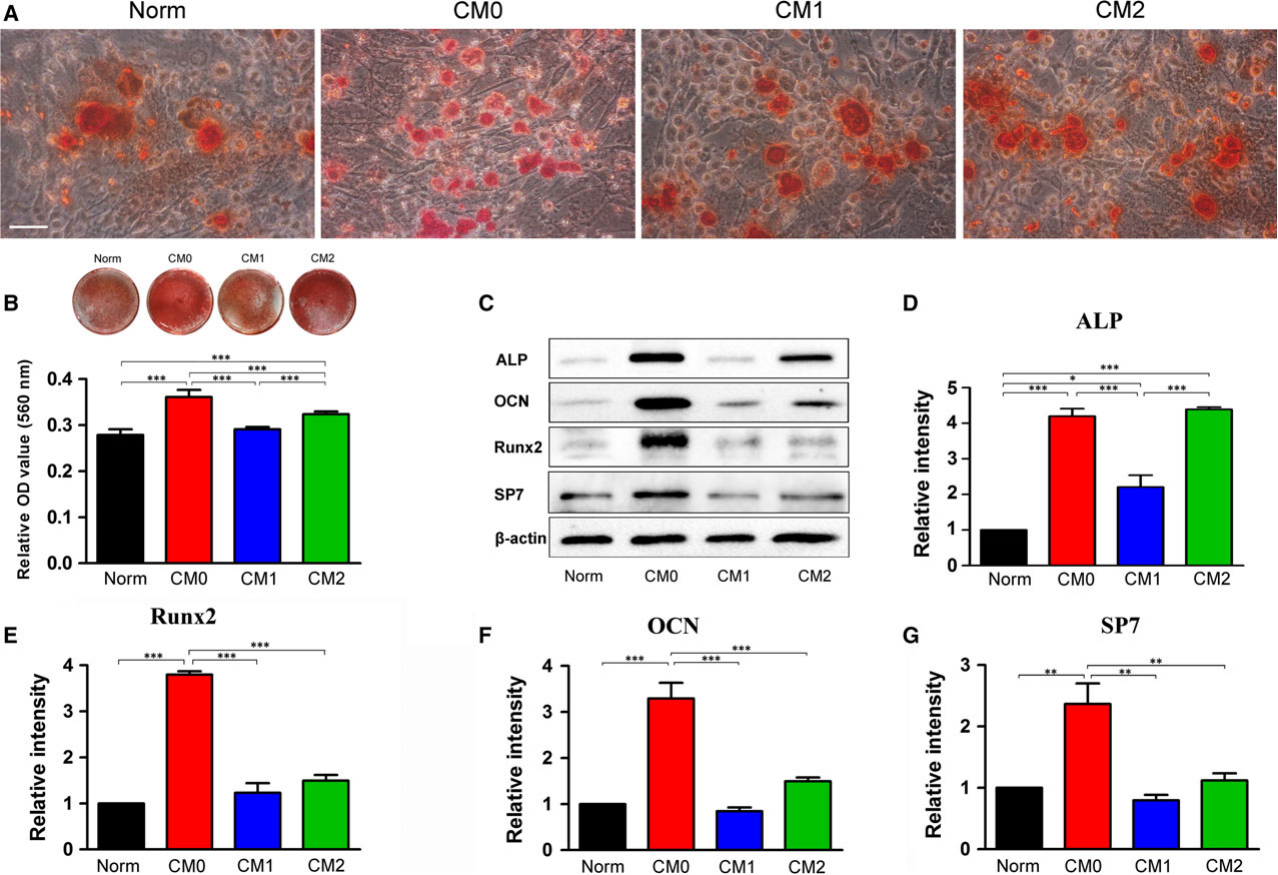
Effects of CMs generated by different Mφ phenotypes on osteogenic differentiation of bone marrow mesenchymal stem cells; Norm was used as a control. (**A**) Representative images of mineralized nodules stained with Alizarin Red after 14 days of osteogenic induction. Scale bar: 50 μm. (**B**) General view of Alizarin Red staining and quantitative analysis of mineralized nodules. (**C**) Expression of osteogenic proteins by cells cultured in different CM‐based osteoinductive media was determined by Western blotting. Quantitative analysis of osteogenesis‐related proteins after 7 days of osteogenic induction: (**D**) ALP, (**E**) Runx2, (**F**) OCN and (**G**) SP7. The analysis was based on mean grey values and normalized to β‐actin. Data are expressed as the mean ± SD; *P < 0.05, **P < 0.01 and ***P < 0.001 represent significant differences between the indicated columns.

The osteogenic potentials of BMMSCs induced in CM‐based inductive media generated by unpolarized Mφs (CM0) or polarized Mφs (CM1 or CM2) were evaluated by Alizarin Red S staining and osteogenesis‐related protein expression, with Norm used as the control. BMMSCs under all tested conditions exhibited the potential to undergo osteogenic differentiation and form microscopic Alizarin Red‐positive mineralized nodules (Fig. 4A). Cells induced in CM0‐based osteogenic medium appeared to form more calcium deposits than those induced in CM1‐based or Norm‐based osteogenic medium, as based on Alizarin Red staining (Fig. 4A). Accordingly, quantitative analysis showed that BMMSCs induced in CM0‐based osteogenic medium formed the largest amount of mineralized nodules among all tested groups (*P* < 0.001, Fig. 4B). In addition, the quantity of mineralized nodules in BMMSCs induced under CM2‐based conditions was significantly higher than that induced in CM1‐ or Norm‐based inductive medium (*P* < 0.001, Fig. 4B). Similarly, Western blot analysis revealed significantly higher osteogenesis‐related protein expression (Runx2, OCN and SP7) in BMMSCs induced in CM0‐based osteogenic medium compared with those induced in CM1‐, CM2‐ or Norm‐based osteogenic medium (*P* < 0.01 or 0.001, Fig. 4C, E–G). Moreover, BMMSCs induced in CM0‐ or CM2‐based osteogenic medium expressed significantly higher levels of ALP protein than those induced in CM1‐ or Norm‐based osteogenic medium (*P* < 0.001, Fig. 4C and D). Significantly increased ALP expression in BMMSCs induced with CM1‐based medium was also observed compared to cells induced with Norm‐based medium (*P* < 0.05, Fig. 4D).

**Figure 5:**
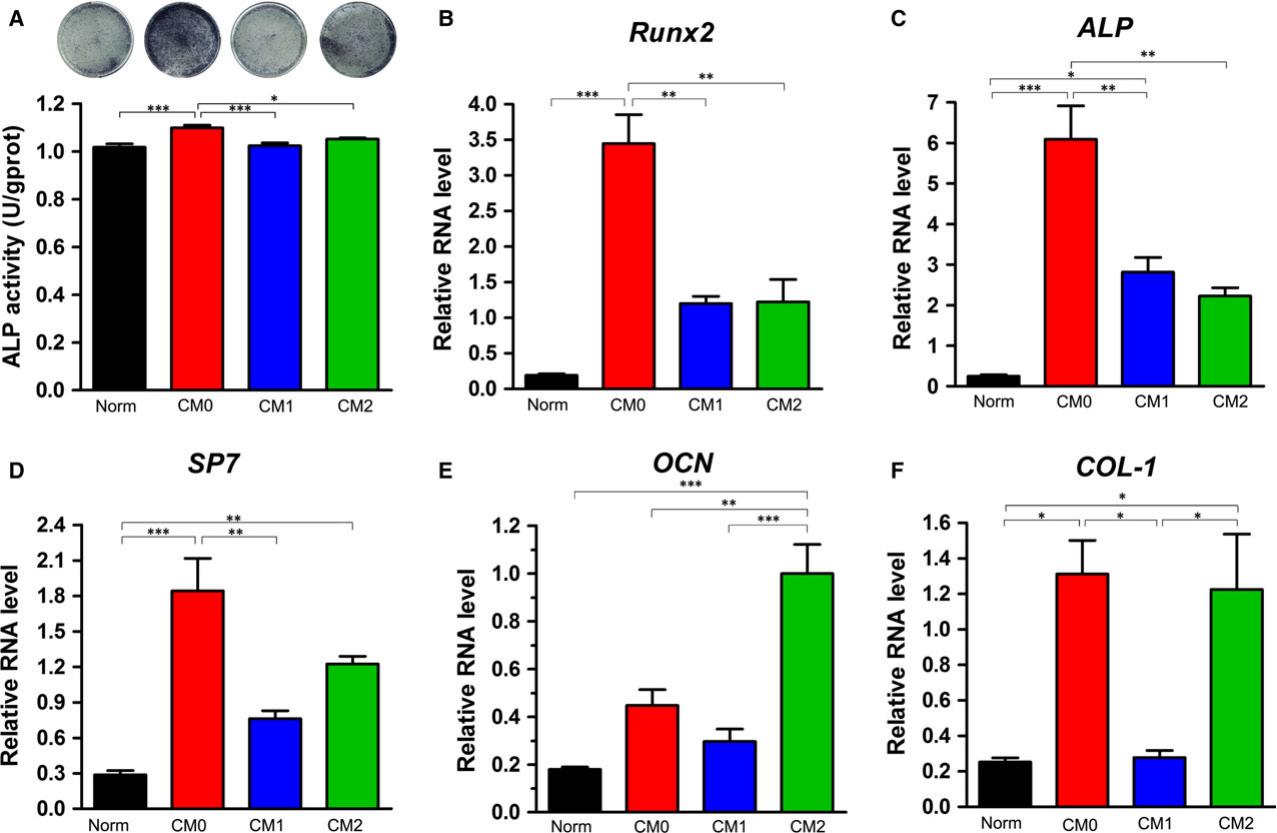
ALP staining and osteogenesis‐related gene expression in bone marrow mesenchymal stem cells induced in CM‐based osteogenic medium for 7 days; Norm was used as a control. (**A**) General view of ALP staining and quantitative analysis of ALP activity. Relative mRNA expression levels (normalized to β*‐actin*) of osteogenesis‐related genes: (**B**) *Runx2*, (**C**) *ALP*, (**D**) *SP7*, (**E**) *OCN*, and (**F**) *COL‐1*. Data are expressed as the mean ± SD; *P < 0.05, **P < 0.01 and ***P < 0.001 represent significant differences between the indicated columns.

The osteogenic ability of BMMSCs induced in CM‐ or Norm‐based osteogenic medium was further evaluated by ALP staining, ALP activity and qRT‐PCR assays. Consistent with Alizarin Red staining, more ALP‐positive cells were observed in CM0‐ or CM2‐based osteogenic medium cultures (Fig. 5A). ALP activity in BMMSCs induced in CM0‐based medium was significantly higher than that of BMMSCs induced in CM1‐, CM2‐ or Norm‐based osteogenic medium (*P* < 0.05 or 0.001, Fig. 5A). Accordingly, the mRNA expression levels of osteogenesis‐related genes (*Runx2, ALP, SP7* and *COL‐1*) in BMMSCs induced in CM0 were significantly higher than in cells induced in CM1‐ or Norm‐based osteogenic medium (*P* < 0.05 or 0.01, Fig. 5B and C). Expression levels of *ALP* and *Runx2* with CM0 induction were significantly higher than with CM2 induction (*P* < 0.01, Fig. 5B and C). However, there was a significant difference in expression of *OCN* genes between cells induced in CM0‐based and in CM2‐based media (P < 0.001, Fig. 5E). Moreover, BMMSCs cultured in CM2 exhibited significantly higher expression levels of *OCN* and *COL‐1* genes compared with cells cultured in CM1‐ or Norm‐based medium (*P* < 0.05, 0.01 or 0.001, Fig. 5E and F).

### Effects of polarized Mφ CMs on the morphology and ECM production of BMMSC sheets

**Figure 6:**
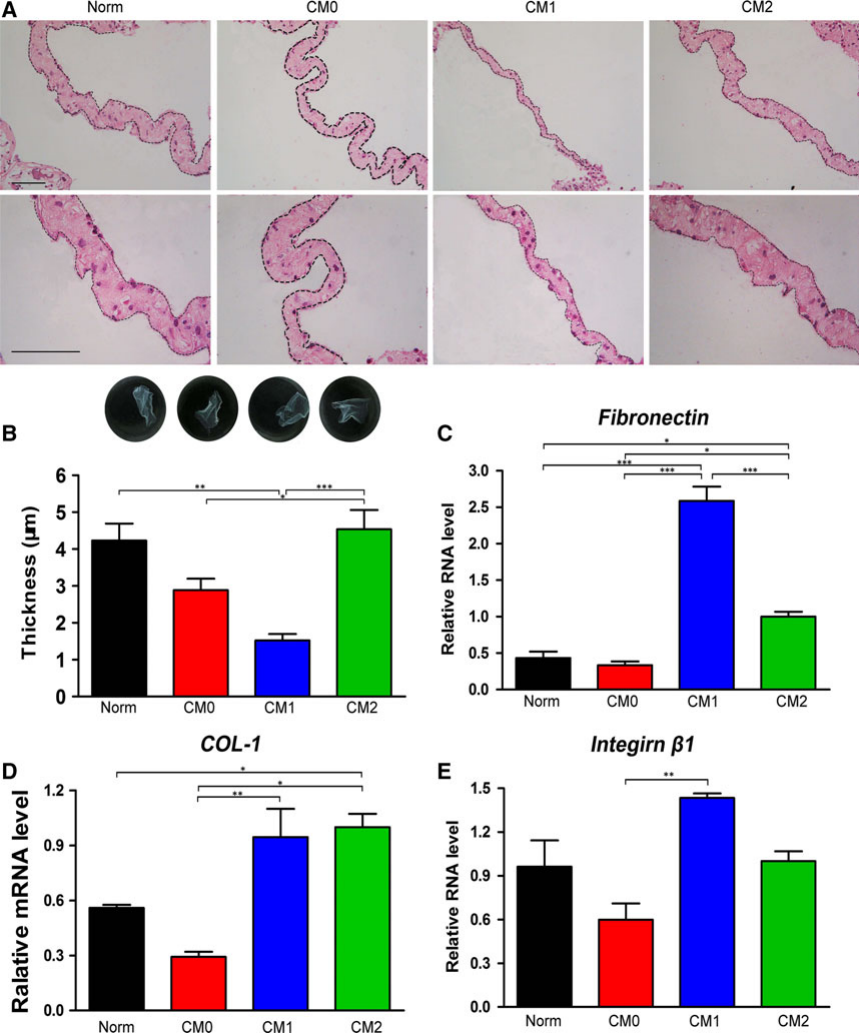
Effects of CMs generated by different Mφ phenotypes on cell sheet formation by bone marrow mesenchymal stem cells; Norm was used as a control. (**A**) Morphologies of cell sheets observed by HE staining following 7 days of sheet induction. Scale bar: 10 μm. (**B**) Representative images of the obtained cell sheets (general view) and quantification of cell sheet thickness using Adobe Photoshop CS5. Relative mRNA levels of extracellular components in the cell sheets (normalized to β*‐actin*): (**C**) *fibronectin*, (**D**) *COL‐1* and (**E**) *integrin* β*1*. Data are expressed as the mean ± SD; *P < 0.05, **P < 0.01 and ***P < 0.001 represent significant differences between the indicated columns.

After 7 days of induction, all cells in the four tested groups had formed ivory and membrane‐like cell sheets and could be completely detached from the culture plates (Fig. 6B). Differences in the continuity, thickness and cell number of the cell sheets formed in different CM‐based cell‐sheet formation media were evaluated by HE staining. HE staining images of lateral sections revealed that the cell sheets generated in CM1‐based inductive medium had poorer continuity and fewer layers of cells compared with those generated in CM2‐ or Norm‐based medium (Fig. 6A). Quantitative analysis of the cell sheets also revealed that those induced in CM2‐based medium were significantly thicker than those induced in CM1‐ or CM0‐based cell sheet‐inductive medium (*P* < 0.01 or 0.001, Fig. 6B). The thickness of cell sheets generated in Norm‐based inductive medium was significantly higher than the thickness of those generated in CM1‐based medium (*P* < 001, Fig. 6B). The expression levels of ECM‐related mRNAs, including *fibronectin*,* COL‐1* and *integrin* β*1*, were determined by qRT‐PCR, with the sheets generated in CM1‐based medium expressing the highest level of *fibronectin* among the four tested groups (*P* < 0.001, Fig. 6C). Expression of *fibronectin* and *COL*‐*1* in the cell sheets induced in CM2‐based medium was significantly higher than in cells incubated in CM0‐ or Norm‐based cell sheet‐inductive medium (*P* < 0.05, Fig. 6C and D). In addition, significant differences in the expression levels of *COL‐1* and *integrin* β*1* were observed between cell sheets induced in CM1‐ and those induced in CM0‐based inductive media (*P* < 0.01, Fig. 6D and E).

**Figure 7:**
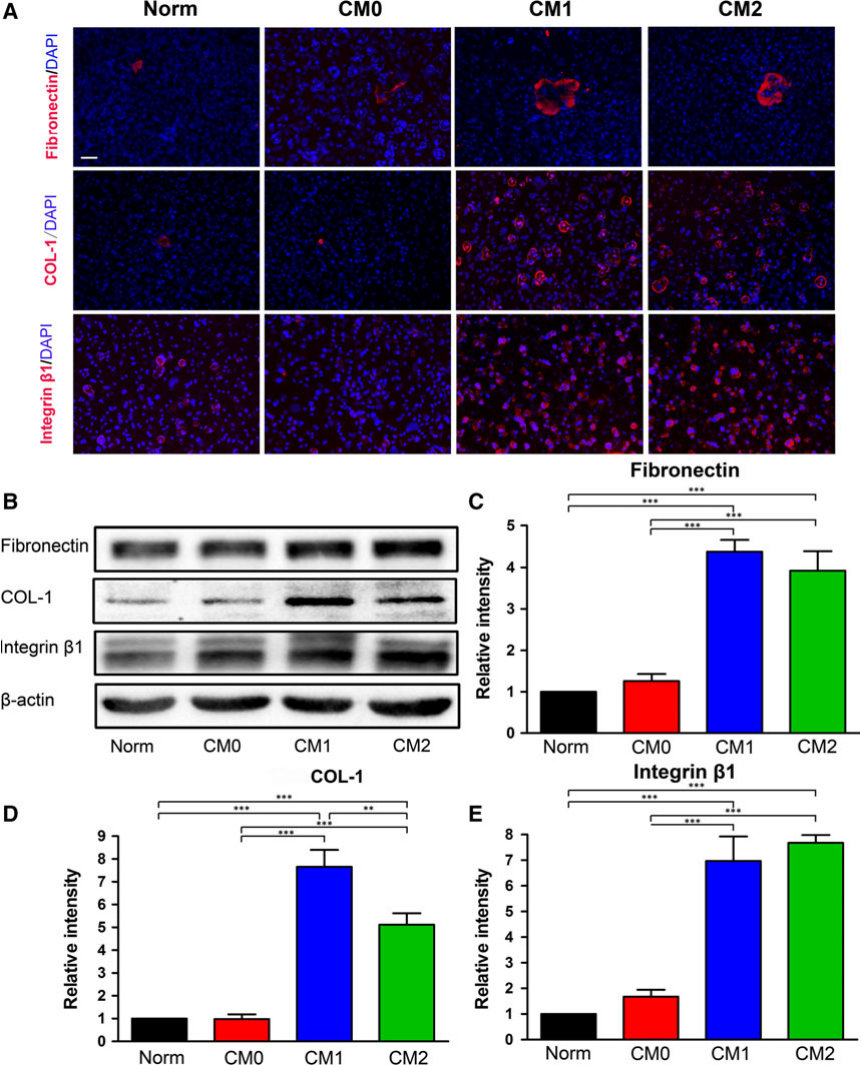
Matrix protein contents in cell sheets were measured by immunofluorescence and Western blotting. (**A**) Representative images of immunofluorescent staining of transverse images of matrix proteins. Extracellular proteins (fibronectin, COL‐1 and integrin β1) are labelled with red fluorescence. Scale bar: 50 μm. (**B**) Western blot analysis of the contents of matrix proteins (fibronectin, COL‐1 and integrin β1) in cell sheets generated by different CM‐based cell‐sheet inductive media after 7 days of induction. Quantitative analysis of the density of protein bands: (**C**) fibronectin, (**D**) COL‐1 and (**E**) integrin β1. The analysis was based on mean grey values and normalized to β‐actin. Data are expressed as the mean ± SD; *P < 0.05, **P < 0.01 and ***P < 0.001 represent significant differences between the indicated columns.

Production of ECM proteins (fibronectin, COL‐1 and integrin β1) in BMMSC sheets was determined by immunofluorescent staining and Western blotting. Consistent with the qRT‐PCR results, immunofluorescent staining of transverse images showed that cell sheets generated in CM1‐ or CM2‐based cell sheet‐inductive medium expressed higher levels of fibronectin, COL‐1 and integrin β1 than those induced in CM0‐ or Norm‐based medium (Fig. 7A). Furthermore, ECM proteins (fibronectin, COL‐1 and integrin β1) were significantly up‐regulated in CM1‐ or CM2‐based medium compared with in CM0‐ or Norm‐based medium, as based on Western blotting (*P* < 0.001, Fig. 7B–E). In addition, cell sheets generated in CM1‐based inductive medium exhibited significantly higher expression of COL‐1 than those generated in CM2‐based medium (*P* < 0.01, Fig. 7D).

## Discussion

In this study, we explored the effect of CMs derived from polarized Mφs on proliferation, differentiation and ECM production of BMMSCs. Murine‐derived Mφ RAW 264.7 cells are a mouse leukemic monocyte macrophage line induced by intraperitoneal injection of Abselon leukaemia virus, which have been widely used as mature Mφs alone without co‐administration of any activators (*e.g*. M‐CSF and GM‐CSF) and do not require monocyte involvement 36. Based on our experience, the use of RAW 264.7 cells allows for reproducible results with manageable effort and is therefore suitable for initial screening. To minimize the immunoregulatory properties of BMMSCs towards Mφs, which can alter Mφ phenotypes, CMs derived from polarized Mφs were used. Most importantly, stimulating factors (*e.g*. LPS plus IFN‐γ applied for M1 Mφ induction and IL‐4 for M2 Mφ induction) were completely removed before collection of CMs. Considering that nutrients in the supernatant of Mφs may be partly consumed, CMs were refreshed every other day during *in vitro* culture. Following induction, specific markers of polarization were evaluated by flow cytometric analysis and qRT‐PCR, both of which showed that Mφs polarized with LPS plus IFN‐γ exhibited highly up‐regulated M1 markers, such as CD86, iNOS, TNF‐α and CCR7, and those polarized with IL‐4 exhibited high expression of M2 markers, such as CD206, Arg and IL‐10 (Fig. 1A). Although flow cytometric data revealed higher CD86 expression for Mϕs polarized with IL‐4 than cells treated with PBS, CD86 expression in Mϕs polarized with LPS plus IFN‐γ was still the highest among all the three tested groups, with up to 95% cells positive for CD86. These findings indicate that unpolarized Mφs were successfully transformed into M1‐polarized and M2‐polarized Mφs. Furthermore, Mφ morphology was observed by immunofluorescence staining under the M1‐polarized (LPS plus IFN‐γ) and M2‐polarized (IL‐4) conditions. It has been reported that M1 Mφs have a pancake‐like shape, whereas M2 Mφs have an elongated cell shape 37. Unfortunately, a similar phenomenon was not observed in the present study. In fact, it is difficult to reach a conclusion regarding the Mφ phenotype and morphology due to differences in both the cell lines and culture systems.

Mφs have been reported to support the survival of and regulate the proliferation of various types of stem cells 21, 22, 23, 24, 25. In the present study, we found that M1‐polarized Mφs exhibited greater capacity to promote proliferation of BMMSCs compared with other tested groups (Fig. 2A). According to CCK‐8 and nuclear EdU incorporation assays, M2‐polarized Mφs were found to impair the proliferative capacity of BMMSCs (Fig. 2A and B). CFU assays also showed that M1 Mφ‐derived CM1 promoted the capacity of cells to form colonies *in vitro* compared with CM0 and CM2 (Fig. 2C and D). However, fewer CFUs were found for BMMSCs incubated in all the Mφ CMs compared with those incubated in Norm medium (Fig. 2C and D). As CM1 contains proinflammatory cytokines derived from M1‐polarized Mφs, the enhanced proliferation of BMMSCs in CM1 is consistent with our previous findings that CM derived from inflammatory stem cells promotes cell proliferation 33. The decreased colony‐forming ability of BMMSCs in CMs compared with those in Norm may, at least in part, be due to a lack of nutrients or a reduction in biological cytokines during the 4‐day incubation period. The different effects of M1‐ and M2‐polarized Mφs on the proliferative ability of BMMSCs have not been previously reported, and the underlying mechanism requires further exploration.

Although extensive evidence has demonstrated that imbalance in M1/M2 Mφs is involved in catabolic remodelling of adipose tissue and contributes to proinflammatory environments that might lead to insulin resistance 36, 38, the effect of polarized Mφs on adipogenic differentiation of BMMSCs has to our knowledge not yet been reported. In the current study, we found that secreted factors derived from M1‐polarized Mφs enhanced the formation of lipid droplets and expression of adipogenesis‐related genes, such as *adiponectin* and *PPAR‐*γ (Fig. 3). Considering that according to previous research, inflammation almost has no effect on adipogenic differentiation of MSCs 33, 39, the up‐regulated adipogenic effect of the CM1‐based inductive medium may originate from non‐inflammatory cytokines derived from M1‐polarized Mφs. However, this phenomenon requires further research. Notably, expression of *adiponectin* (a late marker of adipogenesis) in CM0 was significantly higher than in CM2, but an opposite trend for *PPAR‐*γ expression (an early marker of adipogenesis) was observed in these two groups (Fig. 3D and E). One explanation may be that the optimal conditions for adipogenesis in CM0 and CM2 are different and that BMMSCs cultured in Norm‐ and CM2‐based adipogenic media were undergoing different processes of adipogenesis on day 7. In addition, adipogenic differentiation of BMMSCs in CM2‐based medium occurred later than for cells induced in Norm‐based medium. These findings suggest that M1 Mφs may promote adipogenic differentiation of BMMSCs and that M1 Mφs should be polarized to enhance adipogenesis in Mφ‐focused tissue engineering.

A growing body of evidence has demonstrated that monocytes/Mφs can promote osteogenic differentiation of MSCs, but the number of studies is limited due to a lack of identification of the cell phenotypes used for investigation and because the stimulating factors such as IFN‐γ and LPS used for Mφ polarization, which may interfere with outcomes, were not removed in previous studies 23, 24. Given that MSCs can reprogram Mφs from a proinflammatory M1 phenotype towards an anti‐inflammatory M2 phenotype 27, 28, direct co‐culture of MSCs and Mφs is not recommended when exploring the effect of polarized Mφs on BMMSCs. In this study, both the involvement of monocytes and/or added cytokines and the influence of MSCs on Mφs were avoided by collecting CMs of polarized RAW 264.7 cells. We found that CM0‐based inductive medium significantly promoted osteogenic differentiation of BMMSCs, as measured using Alizarin Red staining and Western blot analysis (Figs 4 and 5). Compared with CM1‐based and Norm‐based inductive media, CM2‐based inductive medium also enhanced the osteogenic capacity of BMMSCs according to Alizarin Red staining, ALP protein expression, and *OCN* and *COL‐1* gene expression (Figs 4B,D and 5E,F). The observed desirable osteogenesis‐promoting effects of M0 Mφs are consistent with previous research, in which Mφs without any stimulation promoted osteogenesis of BMMSCs 24, 25. Furthermore, the positive roles of M2‐polarized Mφs in bone formation are in line with previous research in which biomaterials promoted osteogenesis of human BMMSCs by modulating the macrophage phenotype primarily towards the M2 phenotype 40. However, the effects of M1‐polarized Mφs differ from some previous results, whereby activated inflammatory M1 Mφs were reported to promote induction of osteogenesis in MSCs through oncostatin M production 23. These contradictory data may be derived from the involvement of monocytes and cytokines added in those investigations.

In recent years, cell sheet engineering that generates dense cells, along with their secreted ECM, has been exploited as a promising approach for cell delivery without the need of scaffolding biomaterials 41, 42. In an intact cell sheet, cells reside within their secreted ECM, which serves as a ‘ground substance’ and not only provides physical support for cells and tissue integrity 43, 44 but also protects cell–cell and cell–matrix interactions, which could prevent cell loss, ensure cell survival and facilitate cell engraftment 34, 45. This novel delivery approach enables external cells to be properly engrafted while fully maintaining their bioavailability 32, 46. Given the importance of cell sheets in tissue engineering, we also designed several preliminary tests to assess the effect of polarized Mφs on the secretion profiles of BMMSCs and on their capacity to form sheets. Although all tested groups generated intact sheets, those obtained from cells treated with CM1 were very fragile due to their weak mechanical properties. The lateral section of HE‐stained material also showed that cell sheets generated in CM1‐based medium had poorer continuity and were significantly thinner than those generated in CM2‐based or Norm‐based medium (Fig. 6A and B). Based on qRT‐PCR, immunofluorescent staining of transverse images and Western blotting, we found that CM1‐ and CM2‐based inductive media significantly elevated the abundance of fibronectin, COL‐1 and integrin β1 protein (Figs 6C–E and 7A–E). In our previous study, periodontal ligament stem cell (PDLSC) sheets were manufactured *in vitro* by incubating cells in sheet‐induction media supplemented with various ratios of human platelet lysate (PL) and xenogeneic FBS, and immunohistochemical staining suggested that all resultant cellular materials displayed similar protein profiles of fibronectin, COL‐1 and integrin β1 35. Thus, it appears that immunohistochemical staining of lateral sections is not necessary to evaluate the protein profiles of stem cell sheets. It is therefore we used immunofluorescent staining of transverse sections for investigating related proteins (fibronectin, COL‐1 and integrin β1) involved in cell sheets. Although *fibronectin* and *integrin* β*1* genes (Fig. 6C and E) and fibronectin and COL‐1 proteins (Fig. 7C and D) are expressed at higher levels in the CM1 group, these results do not indicate that cell sheets generated in CM1 contain a greater total ECM content than those generated under other conditions. As clearly shown in Figure 6A and B, the cell sheets generated in CM1 were much thinner than those generated in CM2 and Norm. In fact, it would be difficult to determine the total ECM content in cell sheets generated in the various media using data obtained from our current study because qRT‐PCR, Western blotting and immunofluorescence only assess the tested molecules with the same amount of β‐actin or the same thickness of cell layers. A similar phenomenon was also observed in our previous study in which ‘inflamed’ cells formed thinner cell sheets but appeared to exhibit up‐regulated expression of ECM proteins according to Western blot analysis 34. Thus, we speculate that the effect of CM1‐based cell sheet‐inductive medium on BMMSC sheet formation might be derived from the proinflammatory role of M1‐polarized Mφs, although this possibility requires further research. Regardless, considering both the high production of ECM proteins and the thick cell sheets generated in CM2, it is reasonable to suggest that M2‐polarized Mφs may harness the ECM production ability of BMMSCs *in vitro*, which could enhance the differentiation and regenerative potential of BMMSCs. The assumed outcome should be tested using different *in vivo* models 35.

Taken together, our results indicate that different Mφ phenotypes exert various effects on the proliferation, differentiation and matrix production of BMMSCs *in vitro*. Although CMs generated by M1‐polarized Mφs (CM1) promoted cell proliferation and matrix production, the osteogenic potential of the incubated cells was largely impaired. CM0 enhanced osteogenesis of cells, but the ability of these cells to form sheets was compromised compared to those incubated in CM1 and CM2. In comparison, CM2 not only exhibited the potential to foster osteogenic differentiation of incubated cells, but the cells also displayed a potent capacity to form robust cell sheets. These findings suggest that an appropriate balance of M0, M1 and M2 Mφs would most likely enhance stem cell behaviour and facilitate tissue regeneration.

## Conclusion

The data reported herein demonstrate that different Mφ phenotypes exert different biological effects on BMMSCs. These findings provide the first direct evidence that control of Mφ polarization is an effective strategy for regulating stem cell fate and eventually facilitating wound healing and tissue regeneration. However, the use of CMs does not reflect the overall influences of Mφs on BMMSCs because cellular communication and direct MSC‐Mφ contact may also be of critical importance in final stem cell fate decisions. In addition, it is necessary to identify the essential Mφ phenotype(s) that determine the fate of a specific tissue and to control the balance of each polarized Mφ involved in the wound‐healing cascade. Addressing these questions will allow specific Mφ phenotypes to be induced in the appropriate ratios during the regenerative process, at the specific time points when they are needed, and at the precise locations where they are required, thereby improving the therapeutic outcomes of cell therapy and regenerative strategies.

## Conflict of interest

The authors declare that there is no conflict of interest.
